# Diabetic retinopathy screening and the COVID-19 pandemic in Brazil

**DOI:** 10.5935/0004-2749.20200070

**Published:** 2020

**Authors:** Fernando Korn Malerbi, Paulo Henrique Avila Morales, Caio Vinicius Saito Regatieri

**Affiliations:** 1 Department of Ophthalmology, Universidade Federal de São Paulo, São Paulo, SP, Brazil; 2 Instituto da Visão, São Paulo, SP, Brazil; 3 Tufts Medical School, Boston, Massachusetts, USA

In December 2019, the world became aware of a pneumonia outbreak of unknown origin in Wuhan, China. By January 2020, the Severe Acute Respiratory Syndrome Coronavirus 2 (SARS-Cov-2) virus had been isolated from patients with viral pneumonia^(1)^. Coronavirus disease-2019 (COVID-19) was declared an international health emergency by the World Health Organization on January 30, 2020. On February 26, 2020, the Brazilian Ministry of Health confirmed the first case in Brazil. Two months later, epidemiological models point to a peak that had yet to occur in Brazil.

The coronavirus pandemic focuses on providing medical attention for the affected patients and protecting others from infection, mainly by testing, hygiene, and social isolation. In the meantime, other important health issues are emerging that are related to providing the best care for people with non-COVID-19 related diseases^(2)^.

Ophthalmology has played a central role in the COVID-19 pandemic from the beginning because of the heroic role of Dr. Li Wenliang, a 34-year-old Chinese ophthalmologist whose observations led to the first warning of a possible “SARS-like” epidemic on December 30, 2019. This predated international acknowledgment of the pandemic by more than three weeks. Dr. Li was later was punished by the police for sending warning messages in a chat group about the new coronavirus. On February 7, 2020, he died of COVID-19 in Wuhan Central Hospital^(3)^.

Ophthalmologists are among the groups of physicians with the highest risk of contracting the virus while seeing patients^(4)^ because of their close physical proximity with patients during clinical exams. Among the several ophthalmologic conditions that require timely diagnosis and treatment (and whose detection and treatment has been most affected because of the pandemic), the most important may be diabetic retinopathy (DR), which is considered a major preventable cause of blindness that affects 35% of people with diabetes^(5)^.

Brazil contains the fifth largest population of diabetic individuals worldwide^(6)^; despite the fact that the importance of DR screening and timely treatment has been well established, there is no standardized protocol for DR screening in the Brazilian public health system (Sistema Único de Saúde - SUS). Instead, many patients rely on referrals from family doctors or campaigns provided by volunteers or medical societies. Nevertheless, no previous models are adequate for the “new normal,” and patients with diabetes are at an increased risk of severe forms of COVID-19 and therefore must avoid exposure to the virus; hence, a more rational approach is necessary.

This standardization was badly needed prior to the pandemic because Brazil contains the largest population on the planet with a comprehensive healthcare system that does not require payment by end users; nevertheless, the Brazilian public health system suffers from a chronic shortage of financing^(7)^. With the COVID-19 pandemic, avoiding the unnecessary exposure of diabetic patients and healthcare workers is critical. Furthermore, the demand for DR screening and treatment is expected to surge when contact restrictions are lifted and all patients who could not be screened or treated during the quarantine are once again able to seek medical attention. This unprecedented rise in demand will call for organization and preparedness.

Telemedicine and mobile units are potential alternatives that can provide easier RD screening; there is robust scientific evidence on the safety and efficacy of the telemedicine approach for DR screening in the public health system of Brazil and in other lowand middleincome countries^(5)^. Although it has already been scientifically validated^(8)^, comprehensive implementation lacks a political and regulatory framework. We believe the COVID-19 pandemic offers a historic opportunity to advance this screening system to improve access to ocular health in Brazil and other developing countries.

Brazil has proven itself capable of mobilization in the past - in November 2019, Brazil oversaw the organization of multiple campaigns for the detection and treatment of DR, based on volunteer work, throughout 24 cities in 15 states. The Brazilian ophthalmological commu nity has proven its commitment to the ocular health of the Brazilian people. Faced with this enormous task, we are sure that if the political and regulatory agenda allows, our colleagues will respond appropriately to provide ocular healthcare and to prevent avoidable diabetic blindness in an effective manner, while avoiding unnecessary risks and expenses for both patients and society.
